# Detection and Genetic Environment of Pleuromutilin-Lincosamide-Streptogramin A Resistance Genes in Staphylococci Isolated from Pets

**DOI:** 10.3389/fmicb.2017.00234

**Published:** 2017-02-14

**Authors:** Fengru Deng, Huiwen Wang, Yifei Liao, Jun Li, Andrea T. Feßler, Geovana B. Michael, Stefan Schwarz, Yang Wang

**Affiliations:** ^1^Beijing Key Laboratory of Detection Technology for Animal-Derived Food Safety, College of Veterinary Medicine, China Agricultural UniversityBeijing, China; ^2^Guangdong Provincial Key Laboratory of Protein Function and Regulation in Agricultural Organisms, College of Life Sciences, South China Agricultural UniversityGuangzhou, China; ^3^Institute of Microbiology and Epizootics, Centre for Infection Medicine, Department of Veterinary Medicine, Freie Universität BerlinBerlin, Germany

**Keywords:** PLS_A_ genes, staphylococci, pet, human, multidrug resistance

## Abstract

Increasing emergence of staphylococci resistant to pleuromutilins, lincosamides, and streptogramin A (PLS_A_) and isolated from humans and pets is a growing public health concern worldwide. Currently, there was only one published study regarding one of the PLS_A_ genes, *vga*(A) detected in staphylococci isolated from cat. In this study, eleven pleuromutilin-resistant staphylococci from pets and two from their owners were isolated and further characterized for their antimicrobial susceptibilities, plasmid profiles, genotypes, and genetic context of the PLS_A_ resistance genes. The gene *sal*(A) identified in 11 staphylococcal isolates was found for the first time in *Staphylococcus haemolyticus, Staphylococcus epidermidis*, and *Staphylococcus xylosus*. Moreover, these 11 isolates shared the identical regions flanking the *sal*(A) gene located in the chromosomal DNA. Two *S. haemolyticus* isolates from a cat and its owner carried similar *vga*(A)_LC_ plasmids and displayed indistinguishable PFGE patterns. A novel chromosomal multidrug resistance genomic island (MDRGI) containing 13 resistance genes, including *lsa*(E), was firstly identified in *S. epidermidis*. In addition, *vga*(A)_LC_, *sal*(A), and *lsa*(E) were for the first time identified in staphylococcal isolates originating from pet animals. The plasmids, chromosomal DNA region, and MDRGI associated with the PLS_A_ resistance genes *vga*(A), *vga*(A)_LC_, *sal*(A), and *lsa*(E) are present in staphylococci isolated from pets and humans and present significant challenges for the clinical management of infections by limiting therapeutic options.

## Introduction

Transferable resistance to three chemically distinct classes of antimicrobial agents (pleuromutilins, lincosamides, and streptogramin A; PLS_A_) in staphylococci has been attributed to ABC transporters of the Vga, Lsa, or Sal families. All of the corresponding resistance genes, including *vga*(A), *vga*(A)_V_, *vga*(A)_LC_, *vga*(B), *vga*(C), *vga*(E), *vga*(E)_V_, *lsa*(E), and *sal*(A), were mainly identified in staphylococci from food-producing isolates (Allignet and El Solh, 1997; Haroche et al., 2000; Kadlec and Schwarz, 2009; Jung et al., 2010; Lozano et al., 2012; Hauschild et al., 2012; Wendlandt et al., 2013; Li et al., 2014; Hot et al., 2014). However, the *vga*(A) gene was also identified in *Staphylococcus epidermidis* isolates originating from one cat, clinic environment and an employee (Weiß et al., 2013).

To date, the antimicrobial resistance mechanisms of staphylococci in pets have received less attention than those in food-producing animals. Given the close relationship between pets and their owners, resistant bacteria and their mobile resistance determinants may be exchanged in either direction between pets and humans (Schwarz et al., 2016). The increasingly frequent isolation of methicillin- and multidrug-resistant staphylococci from pets, particularly from dogs with pyoderma and otitis (Cain, 2013), and the occurrence of the same resistance genes in pets and in humans (Butaye et al., 2001; Simjee et al., 2002), may underline the potential role of pets in the transmission of antimicrobial-resistant staphylococci to humans. Currently, there is one published study regarding one of the PLS_A_ genes detected in staphylococci isolated from a cat (Weiß et al., 2013). In the present study, we investigated the distribution of PLS_A_ resistance genes in staphylococci of pet origin and analyzed the locations and genetic environments of these genes.

## Materials and Methods

### Ethics Statement

This research was carried out according to the principles of the Declaration of Helsinki. The involved pet animals were treated with the best practice veterinary care and the informed consent was obtained from pets’ owners. The study protocol was approved by the Ethics Committee of China Agricultural University.

### Bacterial Strains and Antimicrobial Susceptibility Testing

A total of 300 anal swabs or nasal samples were used in this study which were collected from dogs (*n* = 269), cats (*n* = 10), and some of their owners (*n* = 21) in the Veterinary Teaching Hospital of China Agricultural University, Beijing in 2013. The pleuromutilin-resistant isolates were selected by incubation on mannitol salt agar (Luqiao, Beijing, China) supplemented with 2 μg/ml of valnemulin at 37°C for 16–24 h.

The MIC (Minimal Inhibitory Concentration) determinations were done by a standard broth microdilution test following the recommendations of the Clinical and Laboratory Standards Institute [CLSI] (2015). *Staphylococcus aureus* ATCC 29213 was served as the quality control strain.

### Molecular Methods

The genomic DNAs of the valnemulin-resistant isolates were obtained using a Wizard^®^ Genomic DNA Purification Kit (Promega, Madison, WI, USA), and were screened for the *vga, lsa*, and *sal* gene types by PCR assays (Novotna and Janata, 2006; Hot et al., 2014; Li et al., 2014; Wendlandt et al., 2015). Furthermore, the species assignment of the resistant isolates was done by 16S rDNA sequencing and confirmed by MALDI-TOF MS (Bruker Daltonik, Bremen, Germany).

The clonality of the valnemulin-resistant isolates was analyzed by pulsed-field gel electrophoresis (PFGE) as described previously (Wang et al., 2011), and the PFGE results were analyzed using InfoQuestFP software (version 4.5).

The S1 nuclease-PFGE (S1-PFGE) and subsequent Southern blot hybridization were performed to locate the valnemulin resistance genes as described before (Barton et al., 1995). The Low Range PFGE Marker (New England BioLabs, Beverly, MA, USA) served as the size marker.

### Characterization and Sequence Analysis of the PLS_A_ Genes

Genomic DNAs of *S. sciuri* isolate 100N carrying *sal*(A) and *S. epidermidis* isolate 138N carrying *sal*(A) and *lsa*(E) were submitted to high-throughput whole-genome sequencing (WGS), and preceded by library construction on a HiSeq 2500, which produced 150 bp paired-end reads (Berry Genomics Company, Beijing, China).

Draft assembly of the genomic DNA sequences was analyzed by CLC Genomics Workbench 5 (CLC Bio, Aarhus, Denmark). All contigs with the average coverage of >100-fold were searched for the PLS_A_ genes using BLAST analysis. The regions flanking the PLS_A_ genes were identified using *de novo* assembly as earlier described (Zerbino and Birney, 2008) and the random primer walking strategy (Zhang et al., 2009). Sequence analysis was conducted using the ORF Finder (http://www.ncbi.nlm.nih.gov/gorf/gorf.html) and BLAST functions (http://blast.ncbi.nlm.nih.gov/Blast.cgi). To obtain more information about the genetic environments of the PLS_A_ genes in valnemulin-resistant isolates, the random primer walking strategy and inverse PCR were performed.

### Nucleotide Sequence Accession Numbers

The PLS_A_-carrying segments of various isolates in this study have been deposited in GenBank, and their accession numbers are KX712120 (*Staphylococcus haemolyticus* plasmid p131A carrying *vga*(A)_LC_), KX712121 (*S. epidermidis* plasmid p132R carrying *vga*(A)), KX712119 (*S. epidermidis* 138N carrying *sal*(A)), and KX712118 (*S. epidermidis* 138N carrying *lsa*(E)).

## Results

### Characterization of Valnemulin-Resistant Staphylococcal Isolates and Antimicrobial Resistance Profiles

Amongst the 300 anal swabs and nasal samples, 13 (4.3%) valnemulin-resistant isolates with valnemulin MICs of ≥8 μg/ml were detected, which were identified to species level as *Staphylococcus sciuri* (*n* = 8), *Staphylococcus haemolyticus* (*n* = 2), *S. epidermidis* (*n* = 2), and *Staphylococcus xylosus* (*n* = 1) (**Table 1**).

**Table 1:** Characteristics of the 13 valnemulin-resistant staphylococci isolates identified in this study.

Isolate	Species	Host	Origin of samples	Gene	PFGE subtype	Location of PLS_A_^a^	MIC (μg/ml)^b^							
							VAL	TIA	RET	LIN	VIR	SPE	GEN	ERY
139N	*S. sciuri*	Dog	Nasal	*sal*(A)	A	C	**32**	**128**	**16**	**8**	**4**	**≥8192**	1	0.125
140N	*S. sciuri*	Dog	Nasal	*sal*(A)	A	C	**32**	**128**	**32**	**4**	**4**	**≥8192**	1	0.5
145N	*S. sciuri*	Dog	Nasal	*sal*(A)	A	C	**32**	**128**	**32**	**4**	**4**	**1024**	0.5	0.25
90A	*S. sciuri*	Dog	Anal	*sal*(A)	B	C	**64**	**128**	**64**	**8**	**4**	**≥8192**	**64**	0.125
96A	*S. sciuri*	Dog	Anal	*sal*(A)	C1	C	**32**	**128**	**128**	**8**	**4**	**≥8192**	4	0.125
96N	*S. sciuri*	Dog	Nasal	*sal*(A)	C2	C	**64**	**128**	**32**	**8**	**4**	**≥8192**	0.5	**4**
100N	*S. sciuri*	Cat	Nasal	*sal*(A)	D	C	**32**	**128**	**8**	**8**	**4**	**≥8192**	2	0.125
123N	*S. sciuri*	Cat	Nasal	*sal*(A)	E	C	**32**	**64**	**8**	**128**	**4**	**≥8192**	**32**	**128**
131R	*S. haemolyticus*	Human	Nasal	*vga*(A)_LC_	H	P	**16**	**32**	**16**	**16**	**4**	**≥8192**	2	**32**
131A	*S. haemolyticus*	Cat	Anal	*vga*(A)_LC_, *sal*(A)	H	P, C	**16**	**64**	**8**	**16**	**4**	**≥8192**	1	**32**
132R	*S. epidermidis*	Human	Nasal	*vga*(A)	O	P	**16**	**64**	**16**	**64**	**2**	**≥8192**	4	**32**
138N	*S. epidermidis*	Dog	Nasal	*lsa*(E), *sal*(A)	P	C	**>128**	**128**	**128**	**256**	**16**	**≥8192**	**>128**	**>128**
95N	*S. xylosus*	Dog	Nasal	*sal*(A)	n.t.^c^	C	**8**	**32**	**16**	**8**	**8**	**2048**	2	0.125

*^a^The PLS_A_ resistance gene is located on the chromosome (C), on a plasmid (P), or both (P, C). ^b^VAL, valnemulin; TIA, tiamulin; RET, retapamulin; LIN, lincomycin; VIR, virginiamycin M1; SPE, spectinomycin; GEN, gentamicin; ERY, erythromycin. The MICs that classify an isolate as resistant or high MICs (in cases where no applicable breakpoints are available to classify an isolate as resistant) are displayed in bold. *^c^*PFGE was performed only for ≥ two isolates of the same species.*

Nine (eight *S. sciuri* and one *S. xylosus*) of the 13 isolates carried only *sal*(A), one *S. haemolyticus* and one *S. epidermidis* isolate carried *vga*(A)_LC_ and *vga*(A), respectively, one *S. haemolyticus* isolate carried both *vga*(A)_LC_ and *sal*(A), and one *S. epidermidis* isolate carried both *lsa*(E) and *sal*(A) (**Table 1**). Notably, the *lsa*(E) gene was only detected in one *S. epidermidis* isolate of dog origin, and the *vga* genes were detected in staphylococcal isolates originating from both humans and cats. In contrast, *sal*(A) was widespread among the valnemulin-resistant staphylococcal isolates from pets. As the *sal*(A) gene has been previously found exclusively in *S. sciuri* isolates (Hot et al., 2014; Wendlandt et al., 2015), this finding describes for the first time the occurrence of *sal*(A) in *S. haemolyticus, S. epidermidis*, and *S. xylosus*.

All 13 valnemulin-resistant isolates displayed resistance or high MICs to pleuromutilins (valnemulin, tiamulin, and retapamulin), lincomycin, virginiamycin M1 and spectinomycin (**Table 1**).

### Genotyping by PFGE

**FIGURE 1:**
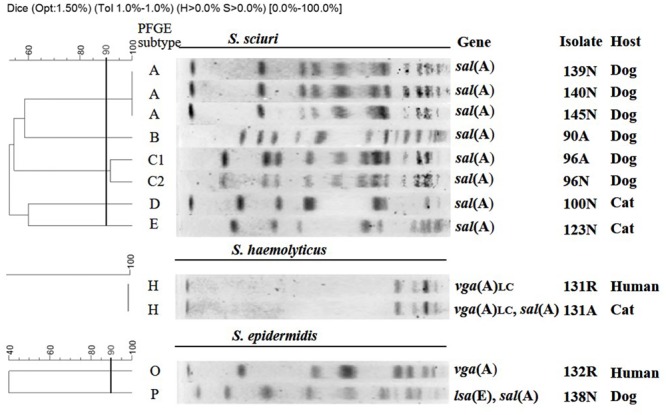
**SmaI-PFGE typing of 12 valnemulin-resistant staphylococcal isolates for which more than a single isolate per species was available**.

Using a cut-off of 90% similarity, the eight *S. sciuri* isolates, which carried highly conserved (>99% nucleotide identity) *sal*(A) genes, clustered into five PFGE types (A–E), with type A accounting for the majority (3/8) of the isolates (**Table 1**; **Figure 1**). Remarkably, the feline *S. haemolyticus* isolate 131A and the human *S. haemolyticus* isolate 131R from the corresponding pet owner, which carried identical copies of *vga*(A)_LC_, showed the same PFGE type H, indicating that they were closely related. This finding supports the idea that these isolates might have been exchanged between the cat and its owner.

### Genetic Environment of PLS_A_ Genes

**FIGURE 2:**
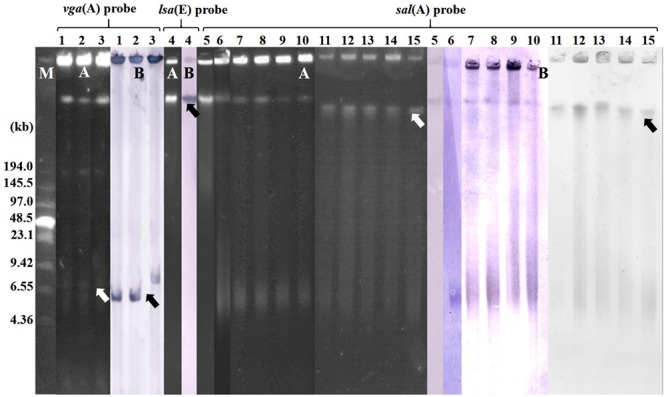
**Localization of pleuromutilins, lincosamides, and streptogramin A (PLS_A_) in the valnemulin-resistant staphylococcal isolates by S1 nuclease-PFGE (A)** and Southern blot hybridization **(B)**. M indicates the Low Range PFGE Marker. The black arrows indicate the location of PLS_A_ gene in Southern blot hybridization and white arrows indicate the position of PLS_A_ gene in genome according to the S1-PFGE and marker. 131A and 131R (lanes 1–2, respectively) were *vga*(A)_LC_-positive *S. haemolyticus* isolates. 132R (lane 3) was a *vga*(A)-positive *S. epidermidis* isolate. 138N (lane 4) was a *lsa*(E)-positive *S. epidermidis* isolate. 90A, 95N, 96A, 96N, 100N, 123N, 131A, 138N, 139N, 140N, and 145N (lanes 5–15, respectively) were *sal*(A)-positive staphylococcal isolates.

S1-PFGE and Southern blot hybridization indicated that all *vga*-type genes in the three isolates were located on plasmids, while all *lsa*(E) and *sal*(A) genes in the respective staphylococci were located in chromosomal DNA (**Table 1**; **Figure 2**).

**FIGURE 3:**
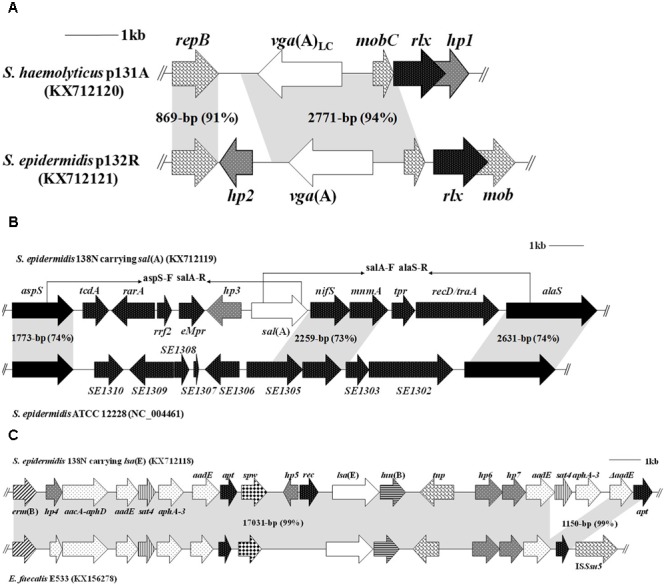
**Comparison of genetic variations of the PLS_A_ genes (*vga*(A)_LC_, *vga*(A), *sal*(A), and *lsa*(E)) of staphylococcal isolates.** Four accession numbers (KX712120, KX712121, KX71211, and KX712118) were newly submitted in this study. The arrows indicate the positions and orientations of the genes. The similarities between the different structures are marked by gray shading. **(A)** Genetic environments of *vga*(A)_LC_ in plasmid p131A from a *S. haemolyticu*s isolate of cat origin and of *vga*(A) in p132R from a *S. epidermidis* isolate of human origin. **(B)** Structure of the novel genetic environment of *sal*(A) in 11 staphylococcal isolates (138N was the representative). The locations of primers used to detect and verify the genetic environment of *sal*(A) in other isolates are indicated by black arrowheads. **(C)** Genetic environment of *lsa*(E) in *S. epidermidis* 138N and structural comparison with the corresponding regions in *E. faecalis* E533.

To determine the regions flanking the *vga*-type genes in isolates 131R, 131A, and 132R, inverse PCRs were performed using the primers vga-F (5′-CAAGCTGAAAAGCCAACAAGG-3′) and vga-R (5′-CCTCGTCAATTTCCCATATAGT-3′), which are located inside the conserved regions of *vga*(A) and *vga*(A)_LC_ genes. Three amplicons of 6056-bp (plasmids p131A and p131R) and 7209-bp (p132R) were obtained (**Figure 3A**). Plasmids p131A and p131R from the feline and human *S. haemolyticus* isolates differed in only nine base pairs, and showed 99% nucleotide sequence identity to plasmid pUR2355 from human *S. aureus* (accession no. JQ312422). Plasmid p132R shared 99% nucleotide identity with the *S. epidermidis* plasmid pUR3036 of cat origin (accession no. JQ312423). The most notable difference between p132R and p131A/p131R was the presence of 13 amino acid substitutions in the Vga protein. Surprisingly, the lincomycin MICs of isolates 131A, 131R, and 132R were 16, 16, and 64 μg/ml, respectively, which is in contrast to the finding that *vga*(A)_LC_ confers higher MICs to lincosamides than does *vga*(A) (Novotna and Janata, 2006).

Genomic DNA of *S. sciuri* isolate 100N carrying *sal*(A) and *S. epidermidis* isolate 138N carrying *sal*(A) and *lsa*(E) were sequenced by WGS. Comparative analysis of the draft genomes of isolates 100N and 138N with the corresponding sequenced genomes of staphylococci from NCBI revealed the presence of a very similar 12.5-kb DNA segment (97% nucleotide identity) containing the *sal*(A) gene inserted between the housekeeping genes *aspS* and *alaS* of isolates 100N and 138N (**Figure 3B**). This DNA segment contains 10 ORFs, of which only *sal*(A) is an antimicrobial resistance gene. Additionally, two pairs of primers, aspS-F (5′-CGTTGTTGAAGATGGTGCGTCT-3′), salA-R (5′-GGACCGAACCTTGAAATGATTG-3′), and salA-F (5′-GATGGATACCTTATAGAAGGTG-3′), alaS-R (5′-GTCTGTATCATAGTTCGTTGG-3′), which are located in the conserved regions of the *aspS, sal*(A), and *alaS* genes (**Figure 3B**), respectively, were designed, and long-range PCRs were preformed to investigate the genetic environments of *sal*(A) in the other nine *sal*(A)-positive staphylococcal isolates (**Table 1**). Two amplicons of 7.4 and 7.7 kb were obtained, and identical sequences were found in all nine isolates.

A 20,154-bp *lsa*(E)-carrying fragment containing 21 ORFs was obtained in the canine *S. epidermidis* isolate 138N (**Figure 3C**) after the conjoint analysis of the results of WGS and modified random primer walking strategy. Similar to the corresponding regions in porcine *E. faecalis* E533 (accession no. KX156278), this *lsa*(E)-carrying fragment also contained the macrolide-lincosamide-streptogramin B resistance gene *erm*(B), the aminoglycoside resistance genes *aacA-aphD, aadE*, and *aphA*-*3*, the spectinomycin resistance gene *spw*, the streptothricin resistance gene *sat4*, and the lincosamide resistance gene *lnu*(B). Further downstream of the *lnu*(B) gene, a second copy of the genes *aadE*-*sat4*-*aphA-3* was detected (**Figure 3C**). This multidrug resistance gene region confers resistance to seven classes of antimicrobial agents and explained why isolate 138N exhibited resistance not only to PLS_A_ antibiotics, but also to spectinomycin, gentamicin and erythromycin (**Table 1**). In addition, the *hp4* gene encoded a predicted protein related to an acetyltransferase enzyme GNAT (GCN5-related N-acetyltransferase, with no confirmed function) from *Enterococcus*.

## Discussion

Increasing emergence of staphylococci that are resistant to PLS_A_ and are isolated from humans and pets is a growing public health concern worldwide. Each of these three classes of antimicrobial agents contains important drugs for human and veterinary medicine. The pleuromutilin antibiotics valnemulin and tiamulin are frequently used in veterinary medicine. Retapamulin was the first pleuromutilin approved for topical use in skin infections of humans caused by staphylococci (Jacobs, 2007). Lincosamides, such as clindamycin and lincomycin, have generally been indicated for the treatment of osteomyelitis but also skin and soft tissue infections caused by staphylococci in humans and pets (Xue et al., 1996; Zetner et al., 2003). Virginiamycin is a two-component streptogramin with a broad spectrum of activity against Gram-positive bacteria. Some antimicrobial agents approved for use in human medicine are also applied to nonfood-producing animals under specific regulations, such as the Animal Medicinal Drug Use Clarification Act (AMDUCA) in the USA. In this study, all 13 valnemulin-resistant isolates displayed resistance or high MICs to pleuromutilins (valnemulin, tiamulin, and retapamulin), lincomycin, virginiamycin M1, indicating that all PLS_A_ genes including *vga*(A), *vga*(A)_LC_, *sal*(A), and *lsa*(E) of these isolates were functional. Interestingly, genes *sal*(A) and *vga*(A)_LC_ coexisted in one feline *S. haemolyticus* isolate 131A, and genes *sal*(A) and *lsa*(E) were co-located in the chromosomal DNA of the canine *S. epidermidis* isolate 138N. Isolate 138N exhibited significant differences in the MICs of valnemulin, lincomycin, and virginiamycin M1 compared with other isolates that carried only one type of PLS_A_ genes (**Table 1**). There have been no studies regarding to *sal, vga*, and *lsa* coexisting in the same isolate, while both *vga*(A) and *lsa*(E) were identified in four bovine staphylococcal isolates (Wendlandt et al., 2015). Notably, *vga*(A)_LC_, *sal*(A), and *lsa*(E) were for the first time identified in staphylococcal isolates originating from pet animals.

The presence of PLS_A_ resistance genes in pet- and human-associated staphylococci indicates the adaptation of these bacteria to antibiotic pressure, given that PLS_A_ resistance genes confer resistance to antimicrobial agents used in both pets and humans. Additionally, *S. epidermidis* 138N carried at least 13 resistance genes conferring resistance to seven classes of antimicrobial compounds (pleuromutilins, lincosamides, streptogramins, macrolides, aminoglycosides, aminocyclitols, and streptothricins), indicating that the use of any of the abovementioned antimicrobial agents may lead to co-selection of the MDR genomic island (MDRGI) in isolate 138N. The plasmids, chromosomal DNA region, and MDRGI associated with the multiresistance genes *vga*(A), *vga*(A)_LC_, *sal*(A), and *lsa*(E) exist in staphylococci isolated from pets and humans, which enhances the dissemination of PLS_A_-resistant staphylococci among pets and humans and presents significant challenges for the clinical management of infections by limited therapeutic options.

The three canine *sal*(A)-positive *S. sciuri* isolates 139N, 140N, and 145N not only shared the identical regions flanking the *sal*(A) gene, but also had the uniform PFGE pattern (subtype A), suggesting that these isolates have originated from a single clone. Remarkably, plasmids p131A and p131R, the two almost identical *vga*(A)_LC_-carrying plasmids present in isolates with the same PFGE subtype (subtype H), were derived from a cat and its owner. This fact suggests that these two isolates might have been exchanged between the cat and its owner due to their extensive contact. In this regard, it is important to consider the current role of dogs and cats as actual family members in many households and, consequently, the close contact to their owners and other family members (Schwarz et al., 2016).

## Conclusion

This is the first description of the PLS_A_ genes in staphylococci of pet origin and also the description of a novel *lsa*(E)-carrying MDRGI in *S. epidermidis*. Moreover, we identified the *sal*(A) gene for the first time in *S. haemolyticus, S. epidermidis*, and *S. xylosus*. Pets are likely reservoirs of antimicrobial-resistant bacteria, warranting the prudent use of all antimicrobials in pet animal medicine.

## Author Contributions

YW and SS designed research; FD, HW, and YL performed research; FD, JL, AF, and GM analyzed data; FD, SS, and YW wrote the paper. All authors listed have approved research for publication.

## Conflict of Interest Statement

The authors declare that the research was conducted in the absence of any commercial or financial relationships that could be construed as a potential conflict of interest.
